# Effectiveness of a Capacity-Building Session for Nursing Students on Account of World No Tobacco Day in India: A Preliminary Study

**DOI:** 10.7759/cureus.86098

**Published:** 2025-06-15

**Authors:** Saudamini G More, Akankshi Bhattacharjee, Sujata Pinge, Supriya S Vyavahare, Laresh N Mistry, Sumeet Agarwal

**Affiliations:** 1 Public Health Dentistry, Bharati Vidyapeeth Deemed to be University Dental College and Hospital Navi Mumbai, Navi Mumbai, IND; 2 Public Health Dentistry, Yogita Dental College and Hospital, Khed, IND; 3 Pediatric and Preventive Dentistry, Bharati Vidyapeeth Deemed to be University Dental College and Hospital Navi Mumbai, Navi Mumbai, IND; 4 Prosthodontics, Bharati Vidyapeeth Deemed to be University Dental College and Hospital Navi Mumbai, Navi Mumbai, IND

**Keywords:** health promotion, oral cancer, paramedical staff, prevention, public health education

## Abstract

Background: Tobacco, being a cash crop in India, its production and consumption is high. Oral cancer, having the prime etiological factor as tobacco, accounts for a substantial number of all cancer cases in India. In order to curb the habit amongst the masses, it is imperative to get the maximum workforce on hand to promote the detrimental effects of tobacco.

Objective: To evaluate the level of knowledge among nursing students about tobacco before and after receiving an intervention in the form of a sensitization lecture about the use of tobacco in India.

Methods: This preliminary study was conducted among a convenience sample of 61 nursing students. A pre- and post-survey questionnaire, comprising demographic details and knowledge about the ill effects of tobacco (eight items), was distributed. The survey questionnaire was tailor-made and validated by five subject matter experts.

Results: The pre-test median was 4.2 (range=1 to 7), and that of the post-test was 4.8 (range=2 to 8). When Student's t-test was applied, a significant difference was observed between the scores (p=0.027).

Conclusion: The level of knowledge was significantly higher after receiving the lecture. Nursing students will be future healthcare professionals. They can act as a channel to educate the population on matters of oral cancer prevention.

## Introduction

In the past few decades, the world has been faced with a burning, or, better yet, a ‘trending’ challenge: tobacco. Tobacco contributes to more than six million deaths a year and can be considered the single largest cause of preventable death globally [1].

Being the world’s second-largest producer of tobacco, India presents a multifaceted issue, impacting economic stability, public health, and societal well-being. The prevalence of tobacco use in various forms, including smoking and smokeless tobacco, has also introduced a complex public health crisis. India has implemented several national-level initiatives, such as the Cigarettes and Other Tobacco Products Act (COTPA), public smoking bans, and widespread awareness campaigns to restrict tobacco use. Promoting tobacco-free institutes/campuses across India was another novel concept [2]. Despite efforts to curb its consumption, tobacco remains widely accessible and culturally embedded, posing obstacles to health promotion initiatives. This persistent issue underscores the urgent need for comprehensive strategies aimed at reducing tobacco consumption, protecting vulnerable populations, and promoting a healthier future for all Indians [3]. Healthcare professionals, particularly nurses, play a pivotal role in tobacco cessation efforts due to their frequent and direct interactions with patients [4]. The World Health Organization (WHO) emphasizes that brief interventions by health professionals can increase tobacco abstinence rates by up to 30%, and nurse-led interventions have been shown to enhance the likelihood of successful quitting by up to 50% [5]. A study done in the southern part of India suggests including a tobacco-control curriculum at the undergraduate level to improve their competency in giving smoking cessation support [6].

World No Tobacco Day, observed annually on May 31st, provides an opportune moment to address these gaps through targeted educational initiatives. Capacity-building sessions tailored for nursing students can enhance their knowledge, attitudes, and self-efficacy regarding tobacco cessation interventions. This analysis on tobacco use motivated us to conduct a capacity-building awareness session on the occasion of World No Tobacco Day, celebrated on 31st May, among students attending a nursing institute. The present pilot study aimed to assess the change in their awareness after the capacity-building session.

## Materials and methods

The present study was an observational cross-sectional pilot conducted on May 31, 2024, at the Bharati Vidyapeeth College of Nursing, Navi Mumbai, India. The Institutional Ethics Committee for Biomedical and Health Research (BEC/2025/523) granted us ethical approval. The present study was a pilot study, and the data were collected as part of a training activity. Ethical approval was obtained for analysis later on. An awareness lecture was arranged for the students attending the Bachelor’s College of Nursing on account of No Tobacco Day. The 20-minute sensitization lecture highlighted the history of tobacco in India, tobacco as a cash crop, the various forms and types of tobacco consumed in India, and the deleterious effects of tobacco in India. The lecture was taken using the didactic method, followed by discussion and a question-and-answer session.

Selection criteria

All participants selected for this study were second and third-year nursing students who consented to participate.

Questionnaire design and validation

A questionnaire was designed in the English language, comprising eight closed-ended questions (multiple-choice questions) (Appendix 1). Face and content validity were assessed by 10 subject experts. Validation scores were calculated based on experts' responses to a structured assessment consisting of eight multiple-choice questions. Each correct response was awarded one point, and incorrect or unanswered questions received zero points. Therefore, the total score for each participant ranged from zero to eight. The scores obtained were converted to percentages. A content validity ratio of 0.80 was obtained, suggesting 80% agreement among the experts. This score was found to be acceptable for using the questionnaire for the study. Internal consistency (reliability) of the questionnaire was assessed by checking the Cronbach's alpha, with values ≥ 0.70 considered acceptable.

The questionnaire was distributed among them before and after the awareness program among the participants. The questionnaire comprised eight items pertaining to knowledge about tobacco consumption in India, health risks related to tobacco, and common cancers related to tobacco use.

A convenience sample of 61 students was considered for the purpose of this preliminary study. The lecture was taken in the nursing college auditorium, and physical questionnaire forms were distributed as a pre- and post-survey. The results of the survey were entered in Microsoft Excel (Microsoft Corporation, Redmond, Washington, United States) and analyzed using IBM SPSS Statistics for Windows, Version 22 (Released 2013; IBM Corp., Armonk, New York, United States). The pre-test and post-test survey responses were categorized as 'correct' and 'incorrect' as percentages and frequencies. The test scores of all of them were calculated out of the total to obtain pre-test and post-test medians and interquartile range. The Wilcoxon signed-rank test (to compare medians of paired samples) was used for conducting inferential statistics (p-value set at 0.05).

## Results

**Figure 1:**
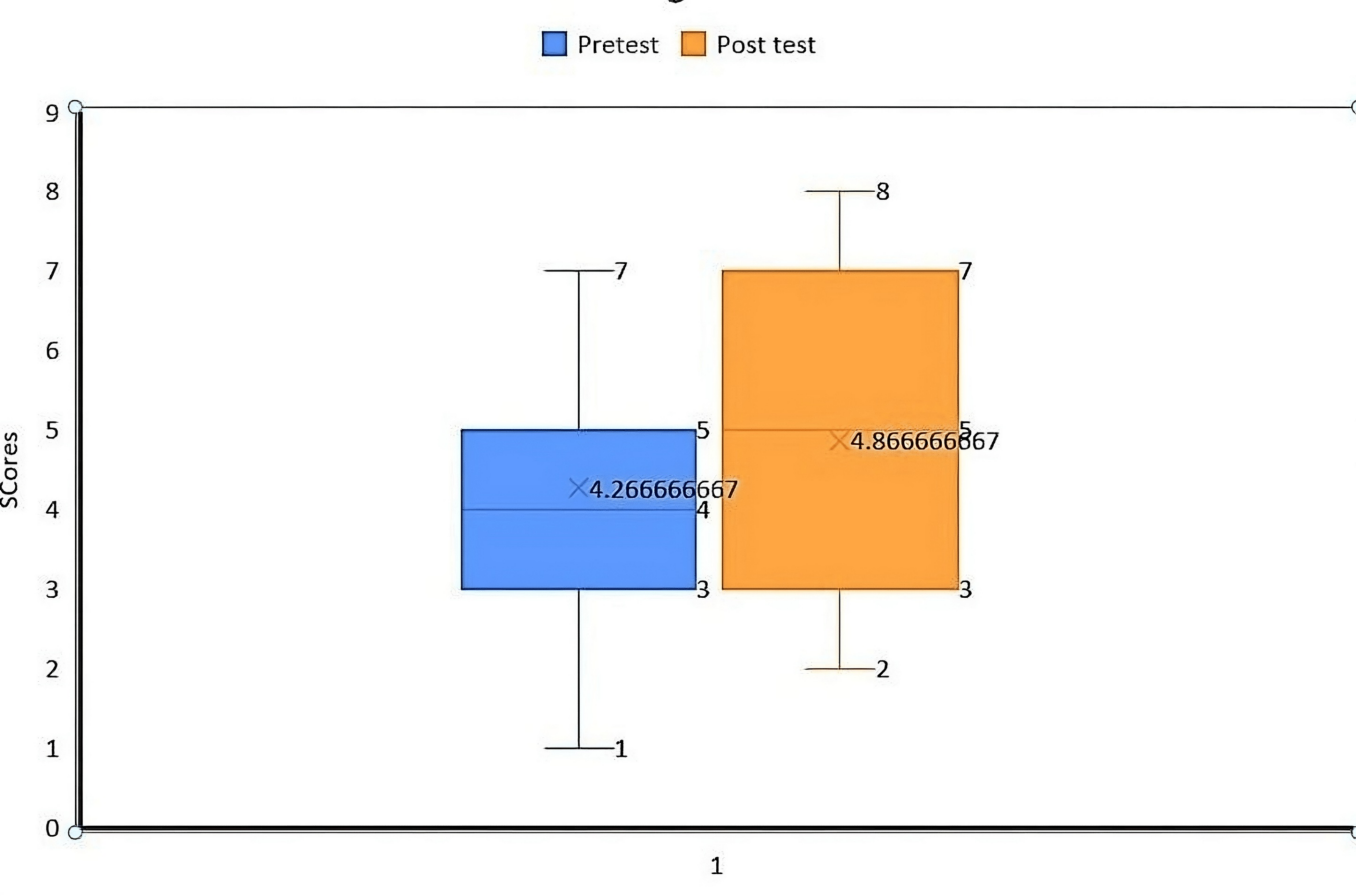
Boxplot depicting the pre-test and post-test scores of participants Test applied: Wilcoxon signed-rank test P-value=0.027

Out of the 61 participants, 39 were females and 22 were males. All participants belonged to the age range of 18 to 21 years. Before the lecture, 13.1% (eight of 61) of the participants and after the lecture, 39.3% (24 of 61) of the participants could answer 75% of the questions correctly. Table 1 shows the participant responses for the pre-test questionnaire, and Table 2 shows the participant responses for the post-test questionnaire. Figure 1 shows the test scores before and after the informative session. The median for pre-test scores was 4.2 (range=1 to 7), while that of post-test scores was 4.8 (range=2 to 8). The difference between the pre-test and post-test scores was significantly different (p=0.027).

**Table 1:** Participants' responses to the pre-awareness program Eight-item questionnaire. Total number of respondents, N=61

Sr No.	Questionnaire	Correct Response N (%)	Incorrect Response N (%)
1.	In terms of tobacco production, India stands at what place worldwide?	21 (34.43%)	40 (65.57 %)
2.	Which type of tobacco use is common among young adults in India?	17 (27.87%)	44 (72.13%)
3.	Which of these is not a health risk due to tobacco use?	21 (34.43%)	40 (65.57%)
4	Which country records the highest number of oral cancer cases?	37 (60.66%)	24 (39.34 %)
5	Which among these is a type of chewing form of tobacco?	29 (47.54%)	32 (52.45%)
6	Answer whether the following statement is true or false: ‘The government bans authorization of tobacco shops in the vicinity of schools/colleges.’	33 (54.10%)	27 (44.26%)
7	Answer whether the following statement is true or false: ‘The government bans smoking in private spaces.’	41 (67.21%)	20 (32.78%)
8.	Which is not a strategy for quitting tobacco?	33 (54.10%)	28 (45.9%)

**Table 2:** Participants' responses to the post-awareness program Eight-item questionnaire. Total number of respondents, N=61

Sr No.	Questionnaire	Correct Response N (%)	Incorrect Response N (%)
1.	In terms of tobacco production, India stands in what place worldwide?	4 (6.3%)	57 (93.7%)
2.	Which type of tobacco use is common in young adults in India?	30 (50%)	31 (50%)
3.	Which of these is not a health risk due to tobacco use?	38 (62.5%)	23 (37.5%)
4	Which country records the highest number of oral cancer cases?	4 (6.3%)	57 (93.7%)
5	Which among these is a type of chewing form of tobacco?	30 (50%)	31 (50%)
6	Answer whether the following statement is true or false: ‘The government bans authorization of tobacco shops in the vicinity of Schools/ colleges.’	46 (75%)	15 (25%)
7	Answer whether the following statement is true or false: ‘The government bans smoking in private spaces.’	26 (43.7%)	35 (56.3%)
8.	Which is not a strategy for quitting tobacco?	46 (75%)	15 (25%)

## Discussion

Capacity-building is defined as the process of developing and strengthening the skills, instincts, abilities, processes, and resources that organizations and communities need to survive, adapt, and thrive in a fast-changing world [7]. With that being known, what capacity-building means in practice remains poorly understood. Capacity-building necessarily means adapting new forms of practice to meet the dynamic or growing nature of needs [8]. Among the many facets of capacity-building, training personnel and collaboration are important ones.

Nurses form an important component of the healthcare chain in society. Often, they are frontline workers in rendering care to patients. In a vast and populous country like India, where a doctor may not be able to reach every individual, nurses can act as an effective workforce to educate people about the ill effects of tobacco [9]. With this notion, an informative session was conducted on account of World No Tobacco Day, and pre- and post-knowledge of nursing students was assessed. 

India's tobacco issue has proven to be exceptionally complex compared to its global counterparts, burdened by a high incidence of tobacco-related diseases and mortality [3]. The 2016-17 Global Adult Tobacco Survey India reported that more than 267 million adults aged 15 and older used tobacco, representing 29% of all adults in the country. Smokeless tobacco is the most common form, with popular products like khaini, gutkha, betel quid with tobacco, paan, and zarda. Tobacco smoking includes bidi, cigarettes, and hookah [10]. Tobacco is known to be the arch criminal for the precancer or cancer of the oral cavity. Approximately 90% of oral cancers of the head and neck region are squamous cell carcinomas of the oral mucosal lining [11]. As an answer to this, significant global efforts have focused on reducing the tobacco epidemic in the last two decades, particularly since the WHO adopted the Framework Convention on Tobacco Control in 2003. By 2015, acknowledging the impact of tobacco use, enhanced tobacco control became a global development goal within the 2030 Agenda for Sustainable Development [12].

The present preliminary research showed that the level of awareness about tobacco use and diseases related to it increased in a single informative session. Around 39% of the nursing students had assimilated an acceptable level of knowledge about the subject in the post-test survey. A study done in Japan showed that nurses could effectively work in tobacco control programs in local setups of the government. The competencies of the public health nurses were driven by motives like strong motivation to pioneer and change tobacco control, unwavering determination to remove barriers to tobacco control, and a strong drive to achieve tobacco control [13]. In a study conducted among Eastern European nurses, the nurses perceived that it was their moral obligation to counsel patients about tobacco cessation. Patients who were asked and counseled by nurses showed a positive interest in quitting tobacco (p <0.001) at three-month follow-up [14]. A study evaluating a customized Tobacco Counseling Training Module (TCTM) for dental students reported substantial enhancements in knowledge, attitude, ability to identify oral manifestations, and self-confidence post-intervention [15]. Similarly, a training workshop at the All India Institute of Medical Sciences (AIIMS), New Delhi, involving healthcare professionals, including nurses and nursing trainees, showed statistically significant improvements in knowledge, attitudes, and skills related to tobacco cessation activities [16]. A systematic review highlighted that smoking cessation training significantly improved nursing students' knowledge, attitudes, and self-efficacy, although changes in actual counseling practices were modest [17].

There were a few studies that reported challenges to this approach. A study assessing Indian nursing students' practices and attitudes towards smoking cessation support revealed that while a majority advised patients about the health effects of smoking, many reported low self-efficacy in delivering cessation support, citing barriers such as lack of training and communication skills. This highlights the necessity for comprehensive training programs that not only impart knowledge but also build confidence and practical skills [6,18].

Innovative approaches like incorporating interactive methods, like role-plays and real patient interactions with appropriate knowledge, can enhance the effectiveness of training programs. Evidence suggests that such experiential learning techniques significantly improve students' counselling skills and confidence [15,19].

The limitation of this preliminary report is that the sample size was small, and the sample belonged to a single nursing institute. Multicentric studies can be planned in the future with a more representative sample. The informative session was of a shorter duration and relied on didactic teaching. There was no follow-up to assess retention.

Future recommendations

Paramedical staff with adequate training and more rigorous capacity-building workshops can be utilized for creating awareness of diseases caused by the tobacco habit. They can also counsel patients at primary health centers and peri-urban and rural clinics with lower doctor-patient ratios.

## Conclusions

In conclusion, the nursing students showed a significant increase in their level of knowledge about tobacco habits and their ill effects on the body. Nurses can be trained for campaigning, health promotion, and tobacco cessation counseling. Nursing students will be future healthcare professionals. They can act as a channel to educate the population on matters of oral cancer prevention. 

The interactive approach and evidence-based content empowered students with the skills and confidence to serve as proactive agents of change in promoting a tobacco-free society. This preliminary report highlights the effectiveness of such initiatives and underscores the need for continued efforts in integrating tobacco control education into nursing curricula.

## Appendices

Appendix 1

Correct answers are highlighted in italics

1.       In terms of tobacco production, India stands in what place worldwide?

a.       1st         *b. 2nd   *         c. 3rd          d. 4th

2.       Which type of tobacco use is common among young adults in India?

*a.       Smoking form*                 b. Chewing form            c. Other forms

3.       Which of these is not a health risk due to tobacco use?

a.       Heart attacks    b. Oral cancer       c. Lung cancer          d.* Liver disease*

4.       Which country records the highest number of oral cancer cases?

a.       China      *b. India   *    c. Pakistan            d. USA

5.       Which among these is a type of chewing form of tobacco?

a.       Paan masala    b. Zarda    c. Mishri        *d. All of these*

6.       Answer whether the following statement is true or false: ‘The government bans authorization of tobacco shops in the vicinity of schools/colleges.’

a.*       True*          b False        c. Not sure

7.       Answer whether the following statement is true or false: ‘The government bans smoking in private spaces.’

a.       True       * b. False*

8.          Which is not a strategy for quitting tobacco?

a.       Counselling      b. Pharmacotherapy        c. Behavioural therapy       *d. Banning tobacco*
